# Association of baseline *ROR1* and *ROR2* gene expression with clinical outcomes in the I-SPY2 neoadjuvant breast cancer trial

**DOI:** 10.1007/s10549-023-06914-2

**Published:** 2023-04-08

**Authors:** Barbara A. Parker, Rebecca A. Shatsky, Richard B. Schwab, Anne M. Wallace, I-SPY 2 Consortium, Denise M. Wolf, Gillian L. Hirst, Lamorna Brown-Swigart, Laura J. Esserman, Laura J. van ’t Veer, Emanuela M. Ghia, Christina Yau, Thomas J. Kipps

**Affiliations:** 1 Department of Medicine and Moores Cancer Center, University of California San Diego, La Jolla, CA 92093 USA; 2 Department of Surgery and Moores Cancer Center, University of California San Diego, La Jolla, CA USA; 3 Quantum Leap Healthcare Collaborative, San Francisco, CA USA; 4 Department of Laboratory Medicine, University of California San Francisco, San Francisco, CA USA; 5 Department of Surgery, University of California San Francisco, San Francisco, CA USA; 6 Center for Novel Therapeutics, University of California San Diego, La Jolla, CA USA

**Keywords:** ROR1, ROR2, Breast cancer, I-SPY2, Outcomes

## Abstract

**Purpose:**

ROR1 and ROR2 are Type 1 tyrosine kinase-like orphan receptors for Wnt5a that are associated with breast cancer progression. Experimental agents targeting ROR1 and ROR2 are in clinical trials. This study evaluated whether expression levels of *ROR1* or *ROR2* correlated with one another or with clinical outcomes.

**Methods:**

We interrogated the clinical significance of high-level gene expression of *ROR1* and/or *ROR2* in the annotated transcriptome dataset from 989 patients with high-risk early breast cancer enrolled in one of nine completed/graduated/experimental and control arms in the neoadjuvant I-SPY2 clinical trial (NCT01042379).

**Results:**

High *ROR1* or high *ROR2* was associated with breast cancer subtypes. High *ROR1* was more prevalent among hormone receptor-negative and human epidermal growth factor receptor 2-negative (HR-HER2-) tumors and high *ROR2* was less prevalent in this subtype. Although not associated with pathologic complete response, high *ROR1* or high *ROR2* each was associated with event-free survival (EFS) in distinct subtypes. High *ROR1* associated with a worse EFS in HR + HER2- patients with high post-treatment residual cancer burden (RCB-II/III) (HR 1.41, 95% CI = 1.11–1.80) but not in patients with minimal post-treatment disease (RCB-0/I) (HR 1.85, 95% CI = 0.74–4.61). High *ROR2* associated with an increased risk of relapse in patients with HER2 + disease and RCB-0/I (HR 3.46, 95% CI = 1.33–9.020) but not RCB-II/III (HR 1.07, 95% CI = 0.69–1.64).

**Conclusion:**

High *ROR1* or high *ROR2* distinctly identified subsets of breast cancer patients with adverse outcomes. Further studies are warranted to determine if high *ROR1* or high *ROR2* may identify high-risk populations for studies of targeted therapies.

**Supplementary Information:**

The online version contains supplementary material available at 10.1007/s10549-023-06914-2.

## Introduction

*ROR1* encodes a developmentally restricted type I receptor tyrosine kinase-like orphan receptor, [1–4] which we identified was a receptor of Wnt5a. [5] ROR1 expression is prominent in embryogenesis, attenuates during fetal development, and is minimal in post-partum tissues. However, ROR1 is expressed by neoplastic cells of many cancer types making it a potential target for cancer therapy [5, 6]. High-level expression of ROR1 on breast cancer cells has been associated with epithelial–mesenchymal transition (EMT), tumor cell proliferation, and metastases [7]. In chronic lymphocytic leukemia (CLL), high-level expression of ROR1 associates with more-rapid disease progression and shorter survival. [8] As such, the expression of ROR1 may have functional significance that can influence clinical outcomes.

*ROR2* encodes another developmentally restricted, type I tyrosine kinase-like orphan receptor that is structurally related to ROR1 and can serve as a receptor for Wnt5a. [9] Recent studies suggest that ROR2 signaling also may contribute to breast cancer progression and/or tissue invasiveness. [10] It is not known whether the expression of *ROR2* correlates with expression of *ROR1*, with specific breast cancer subtypes, or with differences in clinical outcomes.

We examined the relationship between gene expression of *ROR1* and/or *ROR2* and outcomes in breast cancer patients enrolled in the “**I**nvestigation of Serial **S**tudies to **P**redict **Y**our **T**herapeutic **R**esponse with **I**maging **A**nd Molecular Ana**l**ysis 2” (I-SPY2 TRIAL) study. I-SPY2 is an adaptive platform for investigating novel agents for neoadjuvant treatment of high-risk patients with poor prognosis, newly diagnosed early breast cancer. [11, 12] I-SPY2 employs clinical biomarkers to classify patients’ tumors into subtypes, allowing for randomization of patients into groups that can undergo treatment with or without novel agents proposed for neoadjuvant therapy. Pretreatment transcriptome data are collected on each tumor sample, which is annotated with biomarker subtypes into subgroups that have disparate clinical outcomes. These data can inform development of new therapeutics for patients with resistant disease. In this study, we interrogated the I-SPY2 clinical and transcriptome dataset to determine whether the gene expression levels of *ROR1* or *ROR2* at diagnosis, alone or together, correlate with clinical subtype, response to neoadjuvant chemotherapy, or event-free survival (EFS).

Although expression of *ROR1* or *ROR2* transcripts generally correlate with the expression of ROR1 or ROR2 protein [13], studies have identified alternative splice variants of each of these genes that appear unable to encode surface proteins [14], making this correlation tentative. Nonetheless, platforms for interrogating the genes expressed in breast cancers increasingly are being used to identify subtypes of this disease that have prognostic value. We hypothesize that prognostic value also may be observed in stratifying breast cancers with respect to their relative levels of *ROR1* and/or *ROR2* in the context of residual disease or associated clinical subtype.

## Materials and methods

### Patients and the I-SPY2 trial

We interrogated the clinical significance of high-level gene expression of *ROR1* and/or *ROR2* in 989 patients with stage II or III breast cancer and high-risk disease by clinical criteria (HR- HER2- or HER2 +) or high-risk disease according to the 70-gene signature. [15] Patients were enrolled in one of nine completed/graduated/experimental and control arms in the multi-center, multi-arm neoadjuvant I-SPY2 clinical trial (NCT01042379, IND 105,139) as depicted in Supplemental Fig. 1. Detailed descriptions of the I-SPY2 study design, eligibility, and assessments are as reported [16–22].

**Fig. 1:**
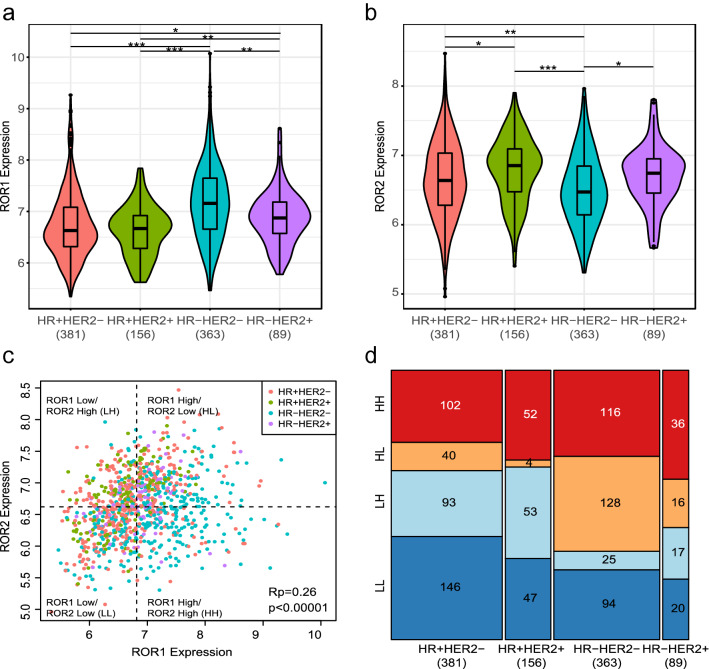
Distribution of *ROR1* and *ROR2* expression by HR/HER2 subtype. **a, b** Violin plots of log2-scaled normalized **a** *ROR1* and **b** *ROR2* expression by HR and HER2 status. Asterisks reflects pairwise Wilcoxon-rank sum test p values (*** *p* < 0.0001, **0.0001 < *p* < 0.001, *0.001 < *p* < 0.05). Color denotes receptor subtype (pink: HR + HER2-, green: HR + HER2 +, aqua: HR- HER2-, purple: HR-HER2) **c** Scatter plot of *ROR2* vs. *ROR1* expression level. Color reflects receptor subtype (pink: HR + HER2-, green: HR + HER2 +, aqua: HR- HER2-, purple: HR- HER2+). Dotted lines indicate median *ROR1* and *ROR2* expression values, which were used to define four patient subsets by dichotomized *ROR1* and *ROR2* expression (*ROR1* above median: *ROR1*-High; *ROR2* above median: *ROR2*-High). **d** Distribution of dichotomized *ROR1/ROR2* expression subsets by HR and HER2 status. Color reflects *ROR1/ ROR2* expression groups (red: *ROR1*-High/*ROR2*-High (HH); orange: *ROR1*-High/*ROR2*-Low (HL); light blue: *ROR1*-Low/*ROR2*-High (LH); blue: *ROR1*-Low/*ROR2*-Low (LL)

### Ethics

Institutional Review Boards at all participant institutions approved the protocol. All patients provided signed informed consent to allow for research on their biospecimen samples in association with clinical outcome data.

### Datasets

Platform corrected, log2-transformed, and normalized gene-level transcriptomic data generated from pretreatment tumor samples assayed on Agilent 44 K expression arrays were obtained from NCBI’s *Gene Expression Omnibus* (GEO) (GSE194040). We obtained patient-level scores from expression signatures reflecting estrogen receptor signaling, HER2 signaling, and proliferation from the supplemental data of the associated publication. [22]

### Statistical analysis

We assessed association between *ROR1* or *ROR2* gene expression levels and hormone receptor (HR) and human epidermal growth factor receptor 2 (HER2) defined subtypes using a Kruskal–Wallis test with post hoc pairwise comparisons by Wilcoxon-rank sum tests with default (Holm) adjustment for multiple hypothesis testing. We used logistic regression to assess association between *ROR1* or *ROR2* expression levels and pathologic complete response (pCR) with significance assessment, using the likelihood ratio test comparing models with or without the biomarker term. We performed analyses with univariate and multivariate models, adjusted for subtype and treatment, conducted within-subtype analyses, with and without adjusting for treatment, as well as exploratory analyses within subtype and within arm. We used multivariate Cox proportional hazard modeling to assess association between *ROR1*/*ROR2* expression levels and EFS with significance assessment, using the likelihood ratio test (comparing models with/without the biomarker term). These analyses were performed in the overall population adjusting for subtype and treatment and extent of residual disease (RCB-0/I vs. RCB-II/III [23]), and among RCB-0/I and RCB-II/III patients, adjusting for subtype and treatment; within-subtype analyses adjusting for treatment, for treatment and extent of residual disease, and among RCB-0/I and RCB-II/III patients. Association between *ROR2* expression levels and subtype, pCR, and EFS were similarly evaluated. Pearson correlation was used to assess correlations between the expression levels of *ROR1* and *ROR2* with expression levels of EMT-related pathway genes, including *Hippo/Yap/TAZ*, *WNT5A, BMl1, BCL2*, and *GLI1*, as well as two ER-related, two HER2-related, and two proliferation-related expression signatures. In addition, we also compared expression levels of these genes and signatures between patient subsets defined by *ROR1* and *ROR2* expression levels (above versus below the median) using the ANOVA F test. All analyses were performed using R version 3.6.3 without adjustment for multiple hypotheses testing.

The analysis reported here is a biomarker study of the gene expression of *ROR1* and *ROR2* leveraging data from the I-SPY2 clinical trial. The patient population, specimen collection, assay methods, and trial design were all previously described, and the sample size could not be changed for this study. REMARK criteria were used to report the data. [24].

## Results

### Expression of *ROR1* and *ROR2* in breast cancer subtypes

We examined the expression levels of *ROR1* at baseline by subtype of breast cancer, Fig. 1a. We observed a wide range of *ROR1* expression levels in all subtypes. We noted HR- HER2- breast cancers expressed the highest levels of *ROR1,* followed by cancers with the HR-HER2 + subtype. HR + HER2- tumors expressed lower levels, which were not significantly different from that of HR + HER2 + tumors. In contrast to what we found for *ROR1*, the expression levels of *ROR2* were lowest in HR-HER2- breast cancers and significantly lower than that found in other subtypes; the highest *ROR2* expression levels were observed in the HR + HER2 + subtype, Fig. 1b. A weak-positive correlation was observed between the expression levels of *ROR1* and *ROR2* , Fig. 1c. When dichotomized at the median expression levels of *ROR1* and *ROR2* and divided into 4 subgroups, we observed an association between the subgroups defined by high-level expression of *ROR1* and *ROR2* in breast cancer subtypes, Fig. 1d. The choice of median cut-point for high- versus low-level expression was based upon our prior study using the median cut-point in analyzing *ROR1* expression in breast cancer datasets before and after neoadjuvant treatment. [25, 26] High-level expression of *ROR1* and *ROR2* was noted in a large percentage of HER2 + specimens, whereas high-level *ROR1* and low-level *ROR2* were more common in HR- HER2- tumors. Consistent with the enrichment for HR- HER2- tumors, the subset with high ROR1 and low ROR2 has the lowest expression levels of ER- and HER2-related signatures and the highest gene expression signatures associated with proliferation, Supplemental Table S1.

### Expression of *ROR1* and *ROR2* and likelihood of pCR

**Fig. 2:**
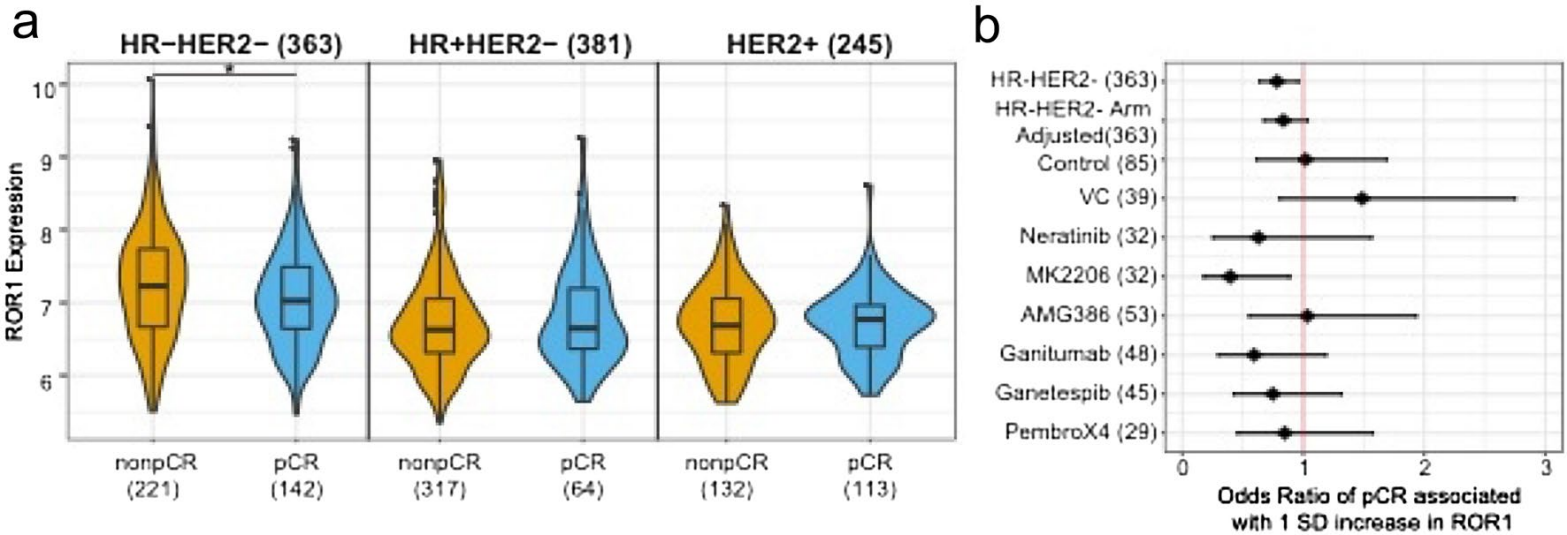
Association between *ROR1* and pCR within HR/HER2 subtypes. **a** Violin plot of *ROR1* expression by pCR and HR, HER2 status. Color reflects pCR status; asterisk denotes likelihood ratio test *p* < 0.05. **b** Forest plot showing odds ratio of achieving a pCR associated with 1 standard deviation increase of *ROR1* expression among HR-HER2- patients (overall, overall adjusting for treatment, and within each treatment arm)

Analysis of likelihood of pCR (RCB-0) [23] by overall population and by subtype revealed a wide range of *ROR1* expression levels within both pCR and non-pCR groups. Although higher expression levels of *ROR1* associated with non-pCR in the HR- HER2- subtype, Fig. 2a, the higher *ROR1* expression observed in non-pCR patients did not retain significance when adjusted for treatment arm. Moreover, there was no apparent association with pCR in other breast cancer subtypes, Supplemental Table S2. Exploratory analysis of pCR in HR-HER2- patients by treatment arm indicated a trend toward negative association of high *ROR1* expression and pCR in 5 of the 8 treatment arms with a notably strong signal in the 32 patients treated on the MK2206 (AKT inhibitor) arm, Fig. 2b. Analysis of *ROR2* expression in relationship to pCR revealed that high-level *ROR2* was not associated with pCR in the overall population or in any subtype, Supplemental Table S2. Therefore, neither high-level *ROR1* nor high-level *ROR2* was associated with the likelihood of pCR.

### Association of *ROR1* / *ROR2* and EFS

**Fig. 3:**
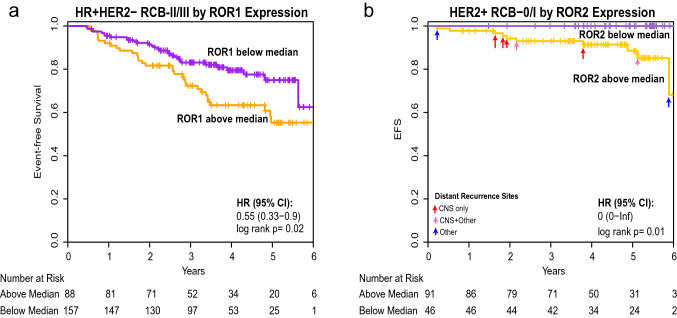
Association between *ROR1* and *ROR2* expression and event-free survival in the context of subtypes and extent of residual disease. Kaplan–Meier plots of **a** HR + HER2- patients with moderate and significant residual disease (RCB-II/III) dichotomized by median *ROR1* expression (purple: below median; orange: above median) and **b** HER2 + patients with no or minimal residual disease (RCB-0/I) dichotomized by median *ROR2* expression (purple: below median; orange: above median)

Breast cancers from patients who had high-level expression of *ROR1* had a worse EFS when adjusted for subtype and treatment arm (HR 1.2, 95% CI = 1.03–1.40, LR*p* = 0.02), Table 1. When assessed in the context of residual disease, high-level expression of *ROR1* associated with a significantly worse outcome for patients with HR + HER2- tumors who had a high post-treatment residual cancer burden (RCB-II/III) (HR = 1.41, 95% CI = 1.11–1.80, LR*p* = 0.01). However, we did not observe a significant association between EFS and *ROR1* among patients with HR + HER2- tumors who had little or no post-treatment residual cancer burden (RCB-0/I) (HR = 1.85, 95% CI = 0.74–4.61, LR*p* = 0.19), which may in part be attributable to a smaller number of events within the RCB-0/I group. We did not observe an association between high-level expression of *ROR1* and worse EFS among patients with HR-HER2- or HER2 + cancer subtypes. Inclusion of *ROR2* in the analysis model did not change these findings. Kaplan–Meier exploratory analysis by *ROR1* above or below the median level indicated that HR + HER2- patients with high-level *ROR1* at baseline and high-tumor burden after treatment (RCB-II/III) had significantly worse EFS, (HR = 0.55, 95% CI = 0.33–0.9, LR*p* = 0.02), Fig. 3a. Kaplan–Meier plots further stratified by RCB class showed that high *ROR1* in HR + HER2- patients with RCB-III had the worst outcome, Supplemental Figure S2a.

**Table 1:** Multivariate cox proportional model of the likelihood of EFS with 1 standard deviation of biomarker expression

		ROR1				ROR2			
		By itself		With ROR2 in model		By itself		With ROR1 in model	
	N	Hazard Ratio	LR p	Hazard Ratio	LR p	Hazard Ratio	LR p	Hazard Ratio	LR p
*Overall Population*									
Adjusting for Subtype and Treatment	905	***1.2 (1.03–1.40)***	***0.02***	***1.19 (1.02–1.40)***	***0.03***	1.09 (0.94–1.27)	0.27	1.03 (0.88–1.21)	0.69
Adjusting for Subtype, Treatment and RCB class	892	***1.21 (1.03–1.41)***	***0.02***	***1.21 (1.03–1.42)***	***0.03***	1.08 (0.93–1.26)	0.33	1.03 (0.88–1.21)	0.67
RCB-0/I Adjusting for Subtype and Treatment	438	1.06 (0.74–1.53)	0.75	0.98 (0.66–1.44)	0.91	1.38 (0.95–2.00)	0.09	1.39 (0.95–2.04)	0.09
RCB-II/III Adjusted for Subtype and Treatment	454	***1.21 (1.02–1.43)***	***0.03***	***1.24 (1.04–1.48)***	***0.02***	1 (0.85–1.19)	0.96	0.93 (0.78–1.11)	0.43

*HR-HER2-*									
Adjusting for Treatment	326	1.06 (0.85–1.33)	0.59	1.07 (0.85–1.34)	0.58	1.00 (0.79–1.27)	0.97	0.99 (0.77–1.26)	0.93
Adjusting for Treatment and RCB class	319	1.07 (0.85–1.35)	0.55	1.08 (0.85–1.36)	0.54	0.98 (0.76–1.27)	0.90	0.97 (0.75–1.26)	0.83
RCB-0/I Adjusting for Treatment	191	0.87 (0.56–1.36)	0.54	0.86 (0.55–1.36)	0.52	1.03 (0.65–1.63)	0.91	1.06 (0.66–1.69)	0.82
RCB-II/III Adjusted for Treatment	128	1.27 (0.97–1.68)	0.08	1.32 (0.98–1.77)	0.07	1.01 (0.76–1.35)	0.92	0.91 (0.67–1.24)	0.55

*HR + HER2-*									
Adjusting for Treatment	359	***1.32 (1.05–1.68)***	***0.02***	***1.33 (1.04–1.71)***	***0.03***	1.08 (0.86–1.37)	0.50	0.98 (0.77–1.26)	0.88
Adjusting for Treatment and RCB class	355	***1.41 (1.11–1.80)***	***0.01***	***1.46 (1.14–1.88)***	***0.005***	1.06 (0.84–1.34)	0.63	0.91 (0.71–1.17)	0.46
RCB-0/I Adjusting for Treatment	110	1.85 (0.74–4.61)	0.19	1.59 (0.57–4.46)	0.38	1.66 (0.72–3.83)	0.23	1.39 (0.55–3.54)	0.48
RCB-II/III Adjusted for Treatment	245	***1.36 (1.05–1.75)***	***0.02***	***1.42 (1.09–1.86)***	***0.02***	1.01 (0.79–1.29)	0.93	0.89 (0.68–1.15)	0.37

*HER2 +*									
Adjusting for Subtype and Treatment	220	1.09 (0.69–1.73)	0.70	0.95 (0.56–1.61)	0.85	1.27 (0.86–1.89)	0.23	1.30 (0.83–2.03)	0.25
Adjusting for Subtype, Treatment and RCB class	218	0.91 (0.57–1.45)	0.69	0.78 (0.46–1.31)	0.34	1.31 (0.88–1.94)	0.18	1.52 (0.96–2.39)	0.06
RCB-0/I Adjusting for Subtype and Treatment	137	0.98 (0.32–3.00)	0.98	0.41 (0.11–1.58)	0.20	***3.46 (1.33–9.02)***	***0.01***	***4.87 (1.57–15.09)***	***0.004***
RCB-II/III Adjusted for Subtype and Treatment	81	0.76 (0.45–1.31)	0.32	0.71 (0.40–1.27)	0.24	1.07 (0.69–1.64)	0.77	1.18 (0.73–1.91)	0.49

*LR p* = Likelihood Ratio *p* value; *N* = number of patients; Bold italics indicates significance by LRp of <0.05

Patients with breast cancers exhibiting high-level expression of *ROR2* did not have a significant difference in EFS compared to patients with tumors with low-level expression of *ROR2* when adjusted for subtype and treatment arm (HR = 1.09, 95% CI = 0.94–1.27, LR*p* = 0.27), Table 1. However, after adjustment for subtype, treatment, and RCB class, patients with HER2 + subtype tumors and minimal residual disease after treatment (RCB-0/I) had significantly worse EFS (HR = 3.46, 95% CI = 1.33–9.02, LR*p* = 0.01,) Table 1, if their breast cancers expressed high levels of *ROR2*. Inclusion of *ROR1* in this analysis model did not change these findings but provided for a numerically larger hazard ratio in the HER2 + RCB-0/I group (HR = 4.87, 95%CI = 1.57–15.09, LR*p* = 0.004). Kaplan–Meier analysis of EFS by *ROR2* above or below the median revealed that, among patients who had little or no residual disease after therapy (RCB-0/I), those with HER2 + tumors and high-level expression of *ROR2* at baseline had a significantly worse EFS than those with HER2 + tumors and low levels of *ROR2* (HR = 0, 95% CI 0-Inf, LR*p* = 0.01), Fig. 3b. Further stratification by RCB class showed that high ROR2 HER2 + patients with RCB-0 or RCB-I had similar EFS, Supplemental Figure S2b. Analysis of EFS by *ROR2* in the RCB-0 (pCR) group with only 6 events did not show a significant difference, Supplemental Fig. 2b. Further exploratory evaluation of 905 I-SPY2 patients with follow-up information regarding recurrence status and site of recurrent disease did not reveal a significant association between high-level expression of *ROR2* and the occurrence of isolated CNS metastases (*N* = 22) or the occurrence of CNS metastases in combination with metastases at other sites (*N* = 18) (data not shown).

### Expression of *ROR1* associates with high-level expression of EMT-related genes

**Fig. 4:**
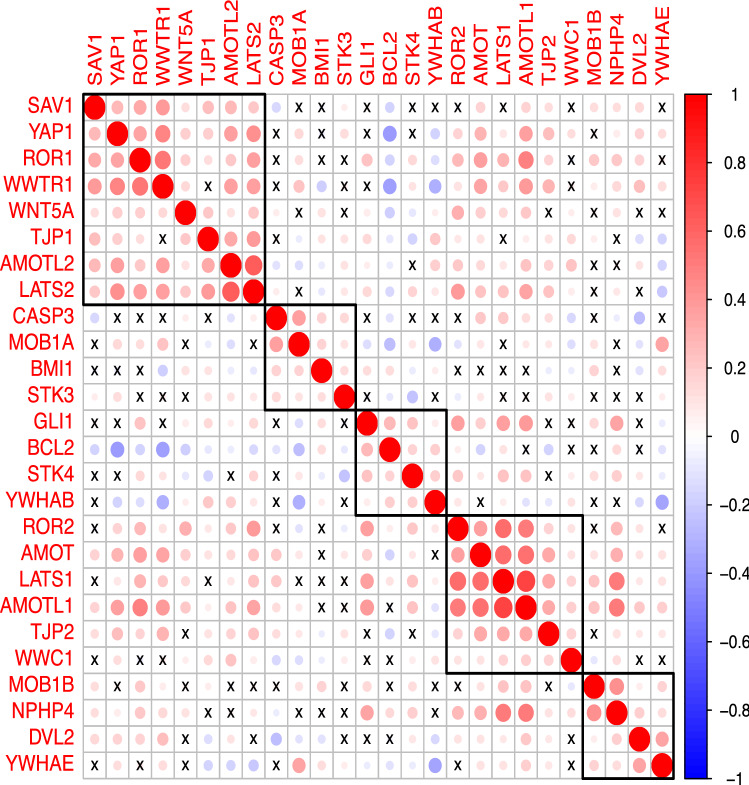
Correlation plot of *ROR1* and *ROR2* expression with EMT-related genes. Genes are organized by hierarchical clustering based on Pearson correlation. Color intensity of the dot reflects the magnitude of Pearson correlation coefficient (red: positive, blue: negative). Size of the dot reflects the p value, and x marks correlations with *p* > 0.05

We performed hierarchical clustering of *ROR1* with 24 EMT-related genes including the Hippo signaling pathway genes from the MSigDB database [27] along with *WNT5a, BMI1, BCL2*, and *GLI1* prompted by associations noted in prior studies on breast cancer or CLL [25, 28–30]. As shown in Fig. 4, we noted a significant association between the high-level expression of *ROR1* and 20 genes evaluated. Eighteen genes each had a positive correlation with *ROR1: WWTR1; AMOTL1; AMOT; LATS2; YAP1; SAV1; LATS1; ROR2; GLI1; AMOTL2; NPHP4; MOB1B; WNT5A; DVL2; TJP2; STK4; MOB1A; and TJP1*. Two genes each had a significant negative correlation with *ROR1*: *BCL2* and *YWHAB*. The strongest correlation between *ROR1* and an EMT-related gene was with *WWTR1* (*TAZ*) (R*p* = 0.54), Supplemental Table S3.

Similarly, 18 EMT-related genes had a positive correlation with *ROR2*: *GLI1; BCL2; STK4; YWHAB; AMOT; LATS1;* *AMOTL1; TJP2; WWC1; NPHP4; DVL2; YAP1; ROR1; WWTR1; WNT5a; TJP1;* *AMOTL2;* and *LATS2*. Only 2 genes had a significant negative correlation with *ROR2*: *MOB1A* and *STK3*. The strongest correlation between *ROR2* and an EMT-related gene was with *LATS1* (R*p* = 0.57), Supplemental Table S3. Consistent with the correlation analysis, comparison of expression levels of EMT genes between the four *ROR1*/*ROR2* groups defined using *ROR1*/*ROR2* expression, 21 of 24 EMT-related genes assessed were differentially expressed between these groups, Supplemental Table S3.

## Discussion

Using the annotated I-SPY2 transcriptome data from a cohort of nearly 1000 patients with newly diagnosed high-risk early breast cancer, we found that high-level pretreatment expression of *ROR1* or *ROR2* had a distinct subtype-specific association with adverse risk. High-level expression of *ROR1* was highest in HR- HER2- subtype and was associated with worse EFS in HR + HER2- patients with high post-treatment residual cancer burden (RCB-II/III). High-level expression of *ROR2* was lowest in the HR- HER2- subtype of breast cancers and higher *ROR2* expression was associated with worse EFS in HER2 + patients with minimal residual disease after therapy (RCB-0/I). High-level *ROR1* or high-level *ROR2* each was associated with high-level expression of genes involved in EMT. Although not correlated with pCR, high-level expression of *ROR1* or *ROR2* distinctly identified breast cancer patients with different tumor subtypes with adverse outcomes. This study highlights the potential prognostic value in assessing the levels of *ROR1* and/or *ROR2* in untreated high-risk early-stage breast cancer and justifies further studies to evaluate the biology and possible value of targeting ROR1 and ROR2 with investigational treatments.

Prior studies from our group showed an association of *ROR1* signaling with stem cell features, EMT, proliferation, and metastases in preclinical models; moreover, the apparent reversal of such features by treatment with an inhibitory anti-ROR1 antibody justified correlative studies of *ROR1* expression with response and clinical outcome [25]. Interrogation of tumor biopsies from 122 patients before and after neoadjuvant chemotherapy revealed the expression level of *ROR1* was increased in residual breast cancer cells after surgery and was associated with enhanced expression of genes associated with EMT, proliferation, and cancer stemness. [25] Therefore, studies of the expression levels of *ROR1 and ROR2* on post-treatment surgical specimens in the I-SPY2 transcriptome dataset, when it becomes available, may provide biologic insights, inform future clinical trials, and determine the optimal tissue and timing for assay.

An exploratory analysis of pCR in HR- HER2- patients by treatment arm indicated a negative trend for the association of high-level *ROR1* with pCR in 5 of the 8 treatment arms with a notably strong signal in the 32 patients treated with MK2206, an AKT inhibitor. This strong signal with MK2206 is not surprising as ROR1 signaling activates the PI3K/AKT/MTOR pathway [25] and high-level expression of *ROR1* may mitigate the anti-tumor activity of an AKT inhibitor in combination with chemotherapy. This observation suggests that investigations of ROR1 blockade with inhibitors of AKT signaling may be informative.

The results for *ROR2* expression significantly extend the prior observations that ROR2 signaling also may contribute to breast cancer progression and/or tissue invasiveness [9, 10]. Studies have shown that ROR2 may regulate the balance of Wnt signaling and cellular heterogeneity during tumor progression. [31] To our knowledge, our study is the first to evaluate the expression levels of *ROR1* and *ROR2* in the same large clinical dataset and to evaluate the association of *ROR2* expression with response and EFS by subtype. Our findings that elevated *ROR2* expression associated with worse outcome in HER2 + patients with minimal post-treatment residual cancer burden (RCB-0/I) was based on a small number of events. As such, analyses of additional datasets are warranted to determine if high-level expression of *ROR2* is associated with adverse outcomes and to determine other factors that may impact outcome in this patient subset.

Our study has several strengths and limitations. Strengths include the fact that the I-SPY2 trial platform includes robust correlative science on serial tumor biospecimens, an active control arm, and contemporary chemotherapy backbone [19]. As a result, we were able to evaluate associations of pretreatment *ROR1* and/or *ROR2* with chemotherapy response by pCR and clinical outcome by EFS. Of note, I-SPY2 eligibility requires that all tumors be clinically or molecularly high risk and patient performance status be excellent. The average age of enrolled patients is more than 10 years younger than that of typical breast cancer patients. [19] Therefore this study may not reflect *ROR1* and *ROR2* expression in the typical patient with breast cancer.

Potential limitations of our study include the analysis of pretreatment tumor specimens only, analysis of gene expression only, and use of Agilent 44 K platform which cannot distinguish between RNA isoforms of *ROR1* or *ROR2* that can or cannot be expressed as cell surface proteins [14]. Additionally, multiple hypothesis testing and small numbers of events in many categories limit statistical power. We analyzed the I-SPY2 transcriptome dataset of baseline pretreatment tumor specimens for expression of *ROR1* and *ROR2* as a transcriptome dataset for post-neoadjuvant surgical tumor tissue is not yet available. Breast cancer biology, hormone receptors, subtype frequency, and mutations can evolve over time under the pressure of systemic therapy; therefore, pretreatment tumor specimens may have different biomarker expressions than post-treatment tumor specimens. [32] However, current biomarkers with clinical utility in early breast cancer are based on assays of pretreatment tumor specimens justifying the current investigation of pretreatment specimens. Future studies of post-treatment surgical specimens, when available, and metastatic specimens are warranted to determine the optimal timing for assessment of *ROR1* and *ROR2* to inform clinical trials of targeted agents.

As noted, the expression or *ROR1* or *ROR2* may not accurately reflect the expression of ROR1 or ROR2 protein. Therefore, we examined the expression of genes that may be upregulated in breast cancer cells through activation of ROR1 or ROR2 signaling. [25, 28] Hierarchical clustering reveals significant associations of *ROR1* with 20 of 24 EMT-related genes, and the strongest association is with *WWTR1* (*TAZ*) a transcriptional coactivator in the Hippo signaling pathway. [33] Similar hierarchical clustering analysis of *ROR2* expression and EMT-related genes reveals significant associations of *ROR2* with 18 of 24 EMT-related genes with the strongest association with *LATS1* (R*p* = 0.57), hypothesized to be a tumor suppressor and the main kinase component in the Hippo signaling pathway [34]. Our analysis showed significant, but variable, correlations between *WNT5a* and *ROR1* or *WNT5a* and *ROR2*. This observation is expected because *WNT5a* gene expression can be modulated by many different pathways [35] and not exclusively by ROR1- and ROR2- regulated pathways. Additional correlation analysis of *ROR1/ROR2* expression groups by median cut-point high/low status with gene signatures revealed that the high *ROR1* and low *ROR2* group enriched for HR- HER2- tumors had the lowest expression levels of ER- and HER2-related signatures and the highest expression levels of proliferation signatures. EMT gene and signature expression that were significantly correlated with *ROR1* and/or *ROR2* expression were also differentially expressed between the four *ROR1/ROR2* defined subsets.

Cancer cells may express ROR1 or ROR2 at levels not observed in normal post-partem tissues and, therefore, the protein antigens encoded by these genes could serve as potential targets for therapy. [25] Our group has developed a humanized monoclonal antibody, cirmtuzumab (now designated as zilovertamab), to ROR1. [36] A Phase 1 study in CLL showed that zilovertamab therapy reversed ROR1 signaling and stemness signatures with minimum apparent toxicity. [37] As a result, zilovertamab is currently under study in CLL and mantle cell lymphoma (NCT03088878) and in advanced breast cancer (NCT02776917) with no additional safety concerns reported to date. [38, 39] Our group has also developed a ROR1 antibody conjugated to MMAE that has been found to be effective in a Richter’s syndrome mouse model [40] and this compound, VLS-101, now zilovertamab vedotin, is under study in hematologic malignancies (NCT03833180) and in solid tumors (NCT04504916). Zilovertamab vedotin was found to have no unexpected toxicities in heavily pretreated patients with lymphoid cancers and to have clinical activity [41]. Other ROR1 [42] and ROR2 targeted therapies are in clinical trials (NCT03504488, NCT03393936).

In summary, we have shown in a cohort of almost 1000 high-risk early-stage breast cancer patients treated on the I-SPY2 platform that pretreatment expression of *ROR1* was higher in HR- HER2- subtype, did not correlate with pCR, and was associated with worse EFS in HR + HER2- patients with high post-treatment residual cancer burden (RCB-II/III). We found that expression of *ROR2* was lowest in HR- HER2- breast cancer, highest in the HR + HER2 + subtype, and did not correlate with pCR. High *ROR2* identified a subset of HER2 + patients who had an excellent response to neoadjuvant treatment (RCB-0/I) but had a higher risk of relapse. Agents targeting ROR1 and ROR2 are in clinical trials and may provide new investigational opportunities. Importantly, these results warrant further studies to determine the value of using high-level expression of *ROR1* and *ROR2* as markers for poor outcome that may inform clinical trials of targeted therapies.

## Supplementary Information

Below is the link to the electronic supplementary material.

Supplementary file1 (DOC 350 KB)

## Data Availability

Platform corrected, log2-transformed, and normalized gene-level transcriptomic data generated from pretreatment tumor samples assayed on Agilent 44 K expression arrays were obtained from NCBI’s *Gene Expression Omnibus* (GEO) (GSE194040). As well, patient-level scores from expression signatures reflecting estrogen receptor signaling, HER2 signaling, and proliferation were obtained from the supplemental data of the associated publication. [22]

## Declarations

### Electronic figure submission

Electronic figures were generated from R version 3.6.3, assembled and formatted using Adobe Illustrator, and embedded in the text body of the manuscript in Word as requested.
