# Supplementary material for: Association of baseline ROR1 and ROR2 gene expression with clinical outcomes in the I-SPY2 neoadjuvant breast cancer trial
Source: Breast Cancer Res Treat. 2023 Apr 8;199(2):281–91. doi: 10.1007/s10549-023-06914-2 (PMC10175386; doi:10.1007/s10549-023-06914-2)
Supplement: Supplementary file 1 — Supplementary file1 (DOC 350 KB) [file 10549_2023_6914_MOESM1_ESM.doc]

# Supplemental Figure S1.

Graduated

Completed In Progress Planned Halted

# Analysis population (989)

DOSTARLIMAB + ORAL PACLITAXEL/ENCEQUIDAR + CARBO DOSTARLIMAB + ORAL PACLITAXEL/ENCEQUIDAR

SYD985 TRILACICLIB

CEMIPLIMAB + REGN3767

CEMIPLIMAB SD-101 + PEMBROLIZUMAB

TUCATINIB DURVALUMAB + OLAPARIB

SGN-LIV1A PEMBROLIZUMAB x 8

PATRITUMAB TALAZOPARIB + IRINOTECAN

PEMBROLIZUMAB x 4 (69)

PLX3397 GANETESPIB (93)

TDM1 + PERTUZUMAB (52)

PERTUZUMAB (44)

MK2206 (94)

GANITUMAB (106)

AMG 386 (134) VELIPARIB + CARBOPLATIN (72) NERATINIB (115)

CONTROL (210)

2010 2011 2012 2013 2014 2015 2016 2017 2018 2019 2020 2021 2022

* Yee et al, JAMA Oncol 2020 Sep 1;6(9):1355-1362.


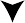

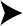


Evaluable for pCR Association (n=989)

Evaluable for EFS Association (n=905)

Missing EFS data as of 2/2019* (n=84)

## I-SPY2 Trial Timeline of Investigational Agents, Number of Patients Enrolled, and Number of Patients evaluable for pathologic complete remission (pCR) and event free survival (EFS).

**Supplemental Figure 2**

**a**

**b**

**Supplemental Fig. 2** Association between ROR1 and ROR2 expression and event-free survival in the context of subtypes and extent of residual disease. Kaplan Meier plots of (a) HR+HER2- patients with moderate (RCB-II) or significant (RCB-III) residual disease dichotomized by median ROR1 expression (purple: below median; orange: above median) and (b) HER2+ patients with no (RCB-0) or minimal (RCB-I) residual disease dichotomized by median ROR2 expression (purple: below median; orange: above median).

**Supplemental Table S1. Association between ROR1/ROR2 defined groups and Proliferation Related, Her2 Related, and ER Related Signatures**.

|  | **ROR1** | | **ROR2** | | **ROR1/ROR2 defined groups** | | | | |
| --- | --- | --- | --- | --- | --- | --- | --- | --- | --- |
|  | Rp | p | Rp | p | Mean normalized expression, HH | Mean normalized expression, HL | Mean normalized  expression, LH | Mean normalized expression, LL | ANOVA F-test p |
| **Proliferation Related Signatures** | |  |  |  |  |  |  |  |  |
| Module11_Prolif_score | 0.12 | 8.29E-05 | -0.21 | 5.77E-11 | -0.15 | 0.42 | -0.24 | 0.04 | 2.60E-11 |
| MP_index | 0.26 | 4.11E-16 | -0.25 | 4.82E-15 | -0.15 | 0.70 | -0.38 | -0.06 | 2.46E-29 |
| **HER2 Related Signatures** | |  |  |  |  |  |  |  |  |
| HER2_Index | -0.13 | 2.47E-05 | 0.16 | 4.80E-07 | 0.11 | -0.29 | 0.30 | -0.11 | 1.03E-08 |
| Mod7_ERBB2 | -0.18 | 5.19E-09 | 0.20 | 8.42E-11 | 0.11 | -0.42 | 0.37 | -0.08 | 1.89E-14 |
| **ER Related Signatures** | |  |  |  |  |  |  |  |  |
| Luminal_Index | -0.36 | 3.29E-31 | 0.05 | 8.95E-02 | -0.14 | -0.60 | 0.43 | 0.25 | 4.40E-29 |
| ER_PGR_avg | -0.25 | 3.06E-15 | 0.30 | 3.46E-22 | 0.12 | -0.77 | 0.53 | 0.02 | 9.93E-40 |

Rp= Pearson correlation coefficient p = p value

H = High expression level of ROR1 or ROR2 above the median L = Low expression level of ROR1 or ROR2 below the median

**Supplemental Table S2. Odds Ratio for Achieving pCR**

|  | **ROR1** | | **ROR2** | |
| --- | --- | --- | --- | --- |
| Overall Population (989) |  | |  | |
|  | Odds Ratio | LR p | Odds Ratio | LR p |
| No covariate adjustment | 1.00 (0.87-1.14) | 0.96 | 0.89 (0.78-1.02) | 0.08 |
| Adjusting for Subtype | 0.88 (0.76-1.03) | 0.11 | 0.88 (0.76-1.02) | 0.10 |
| Adjusting for Subtype and Treatment | 0.94 (0.80-1.09) | 0.40 | 0.92 (0.79-1.06) | 0.25 |
| HR-HER2- (n=363) |  | |  | |
|  | Odds Ratio | LR p | Odds Ratio | LR p |
| No covariate adjustment | ***0.78 (0.64-0.96)*** | ***0.02*** | 0.85 (0.68-1.05) | 0.13 |
| Adjusting for Treatment | 0.84 (0.67-1.03) | 0.10 | 0.86 (0.69-1.09) | 0.21 |
| HR+HER2- (381) |  | |  | |
|  | Odds Ratio | LR p | Odds Ratio | LR p |
| No covariate adjustment | 1.18 (0.89-1.55) | 0.25 | 0.81 (0.63-1.05) | 0.12 |
| Adjusting for Treatment | 1.23 (0.92-1.65) | 0.16 | 0.85 (0.65-1.11) | 0.24 |
| HER2+ (245) |  | |  | |
|  | Odds Ratio | LR p | Odds Ratio | LR p |
| No covariate adjustment | 0.99 (0.70-1.39) | 0.95 | 1.01 (0.76-1.36) | 0.93 |
| Adjusting for Subtype | 0.81 (0.57-1.17) | 0.27 | 1.08 (0.80-1.46) | 0.63 |
| Adjusting for Subtype and Treatment | 0.86 (0.58-1.25) | 0.42 | 1.07 (0.78-1.47) | 0.66 |

Bold italics type indicates significance by LRp of > 0.05.

Odds Ratio for achieving pCR associated with 1 standard deviation increase of biomarker. LR p = Likelihood Ratio p value

# **Supplemental Table S3. Pearson Correlation Coefficients of *ROR1* and *ROR2* Expression with Genes Involved in EMT**

|  | **ROR1** | | **ROR2** | | **ROR1/ROR2 defined groups** | | | | |
| --- | --- | --- | --- | --- | --- | --- | --- | --- | --- |
|  | Rp | p | Rp | p | Mean normalized expression, HH | Mean normalized expression, HL | Mean normalized  expression, LH | Mean normalized expression, LL | ANOVA F-test p |
| **EMT related genes** |  |  |  |  |  |  |  |  |  |
| AMOT | 0.37 | 8.90E-34 | 0.36 | 7.26E-32 | 6.74 | 6.41 | 6.37 | 6.18 | 2.12E-38 |
| AMOTL1 | 0.48 | 2.84E-58 | 0.51 | 3.42E-66 | 6.56 | 6.18 | 6.14 | 5.91 | 5.84E-70 |
| AMOTL2 | 0.22 | 5.71E-12 | 0.21 | 7.07E-11 | 11.93 | 11.82 | 11.70 | 11.53 | 1.42E-08 |
| BCL2 | -0.18 | 1.45E-08 | 0.07 | 3.44E-02 | 8.57 | 8.13 | 8.76 | 8.68 | 1.31E-11 |
| BMI1 | -0.04 | 2.31E-01 | -0.02 | 5.44E-01 | 8.47 | 8.32 | 8.39 | 8.53 | 1.65E-03 |
| CASP3 | 0.01 | 7.27E-01 | 0.04 | 2.35E-01 | 10.50 | 10.43 | 10.44 | 10.47 | 2.79E-01 |
| DVL2 | 0.18 | 2.58E-08 | 0.10 | 1.02E-03 | 8.04 | 8.00 | 7.90 | 7.88 | 4.30E-06 |
| GLI1 | 0.24 | 4.37E-14 | 0.36 | 1.58E-32 | 6.72 | 6.05 | 6.25 | 6.00 | 1.03E-35 |
| LATS1 | 0.28 | 2.52E-19 | 0.57 | 1.19E-84 | 6.12 | 5.58 | 5.85 | 5.52 | 1.98E-63 |
| LATS2 | 0.36 | 3.88E-32 | 0.38 | 8.20E-36 | 8.77 | 8.43 | 8.34 | 8.13 | 4.13E-38 |
| MOB1A | 0.13 | 3.58E-05 | -0.11 | 8.58E-04 | 7.94 | 8.03 | 7.78 | 7.93 | 1.31E-06 |
| MOB1B | 0.20 | 1.76E-10 | 0.05 | 1.44E-01 | 8.85 | 8.79 | 8.63 | 8.72 | 3.97E-07 |
| NPHP4 | 0.21 | 1.62E-11 | 0.28 | 1.05E-18 | 6.80 | 6.57 | 6.62 | 6.51 | 5.14E-16 |
| ROR1 |  |  | 0.26 | 6.42E-17 | 7.39 | 7.47 | 6.45 | 6.30 |  |
| ROR2 | 0.26 | 6.42E-17 |  |  | 7.09 | 6.24 | 6.97 | 6.19 |  |
| SAV1 | 0.33 | 6.58E-26 | -0.04 | 2.05E-01 | 9.68 | 9.81 | 9.33 | 9.42 | 2.85E-23 |
| STK3 | -0.02 | 6.10E-01 | -0.08 | 1.82E-02 | 9.63 | 9.71 | 9.60 | 9.70 | 2.00E-01 |
| STK4 | 0.15 | 1.86E-06 | 0.21 | 1.02E-11 | 8.31 | 8.15 | 8.19 | 8.09 | 3.93E-10 |
| TJP1 | 0.13 | 5.06E-05 | 0.09 | 4.91E-03 | 10.22 | 10.10 | 9.98 | 10.05 | 1.16E-05 |
| TJP2 | 0.16 | 4.23E-07 | 0.18 | 1.66E-08 | 8.04 | 7.91 | 7.89 | 7.81 | 1.39E-08 |
| WNT5A | 0.19 | 3.14E-09 | 0.30 | 3.11E-22 | 8.80 | 8.30 | 8.50 | 8.03 | 6.67E-19 |
| WWC1 | -0.06 | 5.23E-02 | 0.11 | 7.61E-04 | 8.99 | 8.80 | 9.08 | 8.91 | 4.48E-04 |
| WWTR1 | 0.54 | 1.12E-74 | 0.12 | 2.02E-04 | 8.52 | 8.63 | 8.16 | 8.12 | 9.93E-46 |
| YAP1 | 0.35 | 2.66E-30 | 0.17 | 7.56E-08 | 8.26 | 8.24 | 7.95 | 7.85 | 7.34E-21 |
| YWHAB | -0.13 | 2.05E-05 | 0.10 | 1.74E-03 | 13.71 | 13.54 | 13.74 | 13.70 | 8.19E-05 |
| YWHAE | 0.06 | 5.37E-02 | -0.02 | 6.06E-01 | 7.33 | 7.34 | 7.27 | 7.33 | 4.14E-01 |

Rp = Pearson Correlation Coefficient p = p value

EMPTY PAGE LEFT BLANK
